# Co-designing a personalised care plan for patients with rectal cancer: reflections and practical learnings

**DOI:** 10.1186/s40900-024-00553-7

**Published:** 2024-02-08

**Authors:** Grace Gard, Jo Oakley, Kelsey Serena, Karla Gough, Michael Harold, Katya Gray, Helen Anderson, Judi Byrne, Jo Cockwill, Graeme Down, George Kiossoglou, Peter Gibbs

**Affiliations:** 1 Walter and Eliza Hall Institute for Medical Research, WEHI 1 Royal Parade, Parkville, Melbourne, VIC 3052 Australia; 2 Victorian Comprehensive Cancer Council Alliance, Melbourne, Australia

**Keywords:** Consumer involvement, Cancer research, Research design and conduct

## Abstract

**Background:**

Consumer involvement is considered an essential component of contemporary cancer research, with a movement towards participatory methods, to the benefit of consumers and researchers. Overall, in-depth research on participant experiences and perceptions of their co-designer role—and how these may (or may not) change during a co-design project—is limited. The purpose of this paper was to synthesise the reflective accounts of consumers, project staff, and a researcher who partnered on a project to develop a personalised care plan template, with the aim of generating guidance for others looking to partner with consumers in health and medical research. Here, our team of researchers, project staff, and consumers reflect on the experience of working together using Gibbs’ Reflective Cycle, which was completed by team members with responses then undergoing inductive data analysis.

**Results:**

Reflections are categorised under three core themes: (1) setting up the group and building relationships (2) measuring the value of consumer involvement, and (3) potential challenges for consumer involvement. Through reflection on our experiences of co-design, our team developed and identified practical strategies that contributed to the success of our partnership. These include setting expectations as a group; having experienced consumers on the team; having regular, pre-scheduled meetings that run to time; and working to overcome challenges identified by the group such as power imbalances, time commitment, and lack of diversity.

**Conclusion:**

These practical reflections on creating a safe and supportive environment in which genuine consumer involvement can take place could inform other institutions and researchers looking to work meaningfully with consumers in research.

## Abstract (plain language summary)

Consumer involvement in cancer research can inform the initial setting of research priorities, and then the design and conduct of research, with a view to optimising research impact. As part of a project to develop a personalised care plan for patients with newly diagnosed rectal cancer, our team of medical oncologists and project staff partnered with a group of consumers from project initiation. Here, we reflect on our experiences, including the benefits and challenges associated with consumer involvement. Positive aspects of the partnership between consumers, project staff, and the researcher included the establishment of a cohesive team, which substantially improved the study design, conduct, and study outcomes. This experience increased the enthusiasm of the project staff and researcher for consumer engagement in future research. Things that negatively impacted the team included the loss of consumers due to cancer-related health issues. A lack of diversity in the consumer group was recognised as a limitation of the breadth of the consumer voice throughout the project. Upon reflection, there were many important learnings regarding meeting preparation, structure, and team culture that we discuss here, looking to provide practical guidance on optimising consumer involvement.

## Background

Consumer participation is now considered an essential component of cancer research activity. This is reflected in the Australian National Health and Medical Research Council’s (NHMRC) “Statement on Consumer and Community Involvement in Health and Medical Research” (2023) [1]. This states that consumer input to projects should be viewed as a partnership, in which consumers are involved in the development, conduct, and communication of the research. Incorporating the perspective of consumers and their lived experience has the potential to improve the quality and impact of the research, and the transparency and accountability of the research process [2] and is supported by funding bodies and policymakers [3, 4]. Equally, the moral and ethical argument for consumer involvement calls for the democratisation of the research process and suggests the importance of consumer involvement in research that affects them [2]. The goal is that, through working together, clinicians, scientists, and consumers can fashion research projects with a ‘real world’ perspective that can maximally benefit all.

Cancer research creates real world benefits for patients. In 2022 alone, seven new oncology drugs were approved in the USA by the FDA [5]. Cancer research spans across early laboratory-based preclinical work through to large scale phase III clinical trials. Consumers can contribute to all phases of this research.

Our team recently undertook a project to improve education material for patients when they are first diagnosed with rectal cancer. This was prompted by the increasing complexity of multidisciplinary management and the perceived generic “one size fits all” patient support information. Notably, the variable sequencing and combination of surgery, chemotherapy, and radiation therapy as informed by patient and tumour factors, contributes to many potential individualised treatment plans. This RECAP project (A personalised information sheet to improve understanding of a rectal cancer diagnosis and planned management) included contribution from clinical staff (colorectal surgeons, medical oncologists and radiation oncologists), project staff (two project managers and one consumer coordinator) and ‘capable consumers’ familiar with cancer. Cancer Australia define ‘capable consumers’ as consumers who have knowledge from their own experience, and who are able to represent the views of others and ask key questions [6]. Our consumer team was formed under the WEHI Consumer Program with the guidance and contribution of the WEHI Consumer Manager. The WEHI model ‘matches’ highly trained consumers with a researcher or research team, where the personalisation of consumer involvement encourages consumers and researchers to build relationships that go beyond their prescribed roles and individual research projects. Consumers with lived experience of cancer as carers or patients and who expressed an interest in the project were selected by the WEHI Consumer Manager to be part of our team. After working together for over a year on the RECAP project, our team of researchers and consumers is moving towards a model of co-design. Co-design involves consumers, researchers, and project staff working together in partnership as part of an ‘equal team’ to conceive, design, conduct, analyse, and share the outcomes of a series of future research projects.

In reflecting on consumer involvement in the RECAP project, we aimed to:

1. Identify themes from consumer, project staff, and researcher responses.
2. Document and disseminate practical knowledge relating to the team’s experience of consumer involvement in a research project and how this ongoing partnership between consumers and researchers was developed and will be maintained.

## Methods

### The RECAP project

The RECAP project was undertaken at Western Health, Melbourne, Australia. The project’s goal was to develop personalised care plans to improve rectal cancer patients’ understanding of their diagnosis, treatment, and planned follow-up. The project leveraged comprehensive colorectal cancer patient information that is prospectively collected close to real-time in the CRC Registry database [7]. Care plans are personalised by auto-populating a plain language care plan template with key diagnosis, treatment, and follow-up details from the CRC Registry database. The project was funded by the Victorian Comprehensive Cancer Centre (VCCC) and approved by Western Health HREC/23/WH/91656. Consumers who have participated in this project will be remunerated according to the VCCC consumer remuneration policy [8].

Consumers shaped the study design and methodology, making substantive contributions to the development of research materials including the care plan template, patient information sheets and the evaluation survey. Regular team meetings were held throughout the project (six meetings in 2022, four to six projected meetings in 2023). To date, a total of eight consumers have contributed to the project, with the current team comprising four consumers. Contributions made to the care plan template were substantial and worthy of note. Consumers suggested simplified and targeted wording, including the removal of what was considered excess information and confusing diagrams, and condensing the care plan to a double-sided A4 page. Consumer input was also vital to the development of the customised evaluation survey which, after multiple stages of development and review, was streamlined and aligned with current research on the evaluation of implementation effectiveness [9]. Consumer involvement has been reported in this article in accordance with the GRIPP2 Form checklist.

Currently, the RECAP project is at the stage of patient recruitment, whereby patients at Western Health diagnosed with a rectal cancer are offered a personalised care plan from their oncologist that summarises their diagnosis, planned treatment and appointment follow-up schedule. They are asked to complete the evaluation survey when provided with the care plan and will be asked to complete the same survey upon completion of their treatment.

Going forward, meaningful consumer involvement in the project in the form of co-design will be maintained as an ongoing priority. The consumer team will be involved in the development of the interview schedule for semi-structured interviews to be conducted with a purposive sample of patients who receive a personalised care plan. Consumers will be involved in a series of mock-interviews, so that the interview script and procedure can be reviewed and refined. The consumer team will be involved in the planning, development, and reviewing of reports of project findings via peer-reviewed publications or conference presentations, and in less formal means of disseminating results through various consumer networks. Learnings from this evaluation of consumer involvement in the RECAP project thus far will guide this team in all subsequent consumer involvement.

### This evaluation

Use of Gibbs’ Reflective Cycle [10, 11] offered a structured approach to learning from experiences throughout the RECAP project. Gibbs’ Reflective Cycle comprises six stages: description, feelings, evaluation, analysis, conclusions, and action plan. Developed in 1988 this framework has been used to ‘foster self reflection’ across multiple disciplines including within health, encompassing self reflection or group reflection for professional development [12, 13] This framework is suitably broad to capture the experiences and thoughts of consumers, project staff, and researchers and, as a cycle, it lends itself to repeated use at multiple stages. More specifically, the team can return to this cycle, repeating the evaluation, to measure progress and growth in terms of consumer involvement in the project over time. The final stage of the cycle involves reflecting on and developing an action plan to be used throughout the life of the project and to carry into future projects.

Following the consent of consumer participants in the RECAP project to participate in this reflection on project conduct, a two-step process was agreed upon whereby consumers, project staff, and the researcher would first complete Gibbs’ Reflective Cycle. This was either completed electronically or as a pen-and-paper copy. The reflective cycle questionnaire covers questions with free text responses across the six stages as above and is outlined in Table 1 [11]. One member of the project staff and the researcher would then compile the comments and present a draft of the paper to the team to review and approve before submission. In order to create a safe environment for people to express their thoughts, the group decided to make the reflective comments anonymous, such that they were de-identified prior to being shared with the group. Further, it was decided that any comments from the reflection that could appear directly as quotations in this paper would also be de-identified. The consumer team confirmed that this would make for a genuine and inclusive reflection process. The four consumers on the team were invited to be co-authors of this paper in acknowledgement of their ongoing substantive contribution to the project, including the drafting of this paper.

**Table 1:** Gibbs’ reflective cycle questions

*Description*
What did consumer involvement in this project look like (what happened)?
When and where did it happen? Who was present?
What was the outcome of the experience?
Why were you there?
What did you want to happen?
*Feelings*
What were you feeling/thinking during the consumer/researcher interactions?
What were you feeling before and after the interaction?
What do you think about the interaction now?
What do you think other people were feeling about the interaction?
What do you think other people feel about the interaction now?
*Evaluation*
What was good and bad about the experience?
What went well?
What didn’t go so well?
What did you and other people contribute to the situation (positively or negatively?)
*Analysis*
Why did things go well?
Why didn’t things go well?
*Conclusions*
What did you learn from this experience?
How could this have been a more positive experience for everyone involved?
What else could you or others have done?
*Action plan*
If you had to do the same thing again, what would you do differently?
How would you develop the skills required to do this?

An inductive thematic analysis [14] of the responses to the reflection questions was conducted by a member of the project staff and a participating researcher. In this process, common themes were derived from the data such that the themes were data-driven and promoted the development of a grounded theory approach, which aligns with the use of Gibbs’ Reflective Cycle as a tool to learn through and from experience. As these themes were data-driven, they are not necessarily reflective of the researcher or project staff’s own interests or beliefs on the subject, which we believe assists in our attempt to capture an honest reflection on consumer involvement in the RECAP project so far. Four consumers, one clinician and three project staff returned the Gibbs’ Reflective Cycle questionnaire. The responses were de-identified. One project staff member and the researcher familiarised themselves with the data through systematic close reading and re-reading before generating common themes, which were discussed and reworked until consensus was reached. The methodology for data collection and analysis was designed and conducted with the standards of reporting qualitative research in mind [15].

## Results

Reflections are categorised under three core themes: (1) setting up the group and building relationships (2) measuring the value of consumer involvement, and (3) potential challenges for consumer involvement.

### Setting up the group and building relationships

#### Establishing expectations

In their responses to the reflective questionnaire, consumers, project staff, and the researcher highlighted the importance of establishing the expectations for the team, including any interaction between researchers and consumers. Setting up the group involved establishing relationships and roles within the team, which we consider to be an ongoing process that may need to be renegotiated at different points throughout the project’s lifespan. Regardless of the level of consumer engagement in a research project (which the NHMRC Statement explains can span from informing, to consulting, to involvement, to partnership, to consumer-led projects), establishing the role of consumers in the group is an activity that must be done in partnership, so that expectations for the team are clear and aligned.

Nearly all consumers, project staff, and the researcher reflected on the importance of making sure “that expectations are clear at the beginning” (researcher). While expectations of the team were established over time through this project (“I probably should have asked more ‘silly’ questions early on but learnt in time what was required by us all” (a consumer)), reflections also highlighted the benefit derived from having explicit discussion about expectations during the process of setting up the group. One consumer noted “direct discussion about roles of participants at the beginning may have developed confidence more quickly”. These comments indicate the value of an explicit expectation setting activity, such as the development of a vision statement [15]. This process covers the ‘what, why, when and how’ of the project to ensure that everyone understands their role and the roles of others.

Though not explicitly stated, some consumers also indicated a tension inherent in the uncertain expectations of a consumer when participating in research conduct. Consumers are often asked to fulfill two distinct roles: to represent their own lived experience and, concurrently, to act as a voice for the broader consumer community. The responses of the consumers, project staff, and the researcher demonstrated the value of consumers drawing upon their lived experience to provide insight. Yet they also discussed this knowledge as knowledge of “the patient experience” (a project staff member). One consumer took care to draw out this tension, suggesting that they “can’t speak for others”, reiterating that patients, and, indeed, consumers, are not an homogenous group and that there is no singular representative patient experience. These reflections inform the considerable care that must be taken when setting up expectations about the role of a consumer in the research group. Acknowledging that consumer input provides valuable insight into the consumer or patient perspective, while also accepting that individual consumers or small consumer groups should not be asked to speak to a universal patient experience. The researcher on the project captured the importance of keeping sight of this balance: “sometimes consumers speak to their own experience, which may not be reflective of the broader experience of others”.

#### Expertise in the group

As a further consideration in setting up the group, the responses of consumers, project staff and the researcher highlighted the value of having some experienced consumers on the team. These experienced consumers provided guidance and reassurance to the group. One consumer reflected that “the fact that some of us were new to such a task was a bit daunting early on, but having [an experienced consumer] there with all [their] experience in such projects was great”. Having consumers with a great range and depth of experience in the consumer role brings a source of knowledge and perspective to the group. The consumers’ life experiences were also a source of expertise in setting up a successful consumer group. This included previous work that overlapped somewhat with the research activity, with one consumer noting that they “felt comfortable being part of the experience as part of my pre-retirement role was writing care plans”. The researcher offered that “it was good to meet lots of consumers from different backgrounds, also their professional backgrounds were helpful for me, for example a teacher background providing expertise in drafting wording”.

#### Communication and respect

Consumers, project staff, and the researcher commented on the importance of clear, regular, and respectful communication as the foundation for building a trusting relationship and ensuring genuine engagement as a team. One consumer commented that “being often reminded that there were no silly questions or comments was empowering. Furthermore, there was real respect between all attending the meetings, meaning that ideas were listened to and well received then, where necessary, thoughtfully challenged. Over time there was very genuine engagement between staff and consumers”. Another consumer reflected that “communication was effective and there was trust within the group”. We consider this link between communication and trust an essential feature of building consumer engagement into the project; communicating regularly and efficiently, and consistently inviting feedback and comments forms the kind of environment where “a good team was developed as a result of respectful relationships” (a consumer). Practical considerations such as having pre-scheduled meetings with the option to attend remotely via online platforms, being transparent about the purpose of meetings, and ensuring that meetings ran to schedule was reported as contributing to this sense of respect.

### Measuring the value of consumer involvement

Reflections by consumers, project staff, and the researcher on the value contributed by consumer involvement in this project can be organised across four domains: objective value, knowledge/skills gained, attitude change, and affective value.

#### Objective value

Objective value refers to the material and measurable impact of consumer involvement in research. In the case of this project, it refers particularly to the consumer impact on the research design and study documents. Consumers contributed significantly to the design and development of the care plan template, the patient information sheet, and the evaluation surveys. Consumers, project staff and the researcher commented on the substantial and meaningful changes to these documents because of consumer input: the “documents were heavily scrutinised and developed with full consumer input and a variety of voices. Hopefully this has enhanced their quality. They were certainly modified as a result of consumer input” (a consumer); “consumers offered real time feedback about the look and contents of our Personalised Care Plan… I think that the collaboration has made the best personalised care plan possible” (researcher).

#### Knowledge and skills gained

Knowledge and skills gained were highlighted by consumers, project staff, and the researcher as something positive that came out of the consumer involvement in this project. For the project staff and the researcher, gaining knowledge about lived patient experiences, learning how to build trusting relationships within a team, developing practical skills (such as chairing meetings with consumers) and the ability to discuss a research project and its aims in lay terms were all described as outcomes of consumer involvement in this project. A member of the project staff reflected that “the advice given by the consumers felt both invaluable to the project, and also contributed to my understanding of the patient experience”. Practical skills in coordinating a team were acknowledged by the researcher: “I learnt a lot about facilitating meetings and that you can acknowledge everyone’s input, but don’t need to include it all”. Consumers similarly reflected on the knowledge and skills gained through their involvement in this project, with one consumer noting that “consumers have learnt more about research, colorectal cancer and effective communication with researchers”. A consumer also indicated the importance of consumer education and the upskilling that consumers undergo when engaging in a project.

#### Attitude change

Attitude change refers to the profound shift in perspective or priorities for consumers, project staff, and the researcher that occurred over the course of our meetings as a group. Members of the consumer group reported a shift in confidence, leading to a greater understanding and appreciation of what they could offer the project. Consumers reflected on feeling uncertain about the significance of their contribution in the early stages of the project. Yet, their growth in confidence was evident as time went on: “I also was uncertain about what I could contribute, but grew in confidence as we worked more and more on the Project” (a consumer); “I feel that I do have something to offer in such a project” (a consumer).

Equally, some project staff experienced a change in attitude towards consumer involvement in the project over the course of working together as a team. Two members of the project staff reflected on consumer involvement feeling like a barrier to commencing the project: “consumer involvement felt like another ‘hurdle’ or meeting just to get the research going” (a project staff member); “I probably was a bit negative about the worth of consumer involvement before the interaction” (a project staff member). The shift in attitude for these members of the team may be understood as a change from viewing consumer involvement as an external requirement imposed by funding bodies to a process that is integral to the optimal conduct of research, and which is internally demanded by the project itself. After the consumer interaction, project staff reflected that “consumer involvement is valuable and can enhance a project. This is especially important when the project is very patient focussed” (a project staff member); “all patient research should have patient feedback involved at some level” (a project staff member).

#### Affective value

Consumers, project staff, and the researcher reflected on the affective value of consumer involvement in their responses; feelings of gratitude, fulfillment, and recognition recurred across multiple responses. One consumer reflected that “after the interaction fulfilled is how I felt although this possibly sounds a little dramatic—but the session was inclusive of everyone” (a consumer), while other members of the team reported that they “felt it was an overall very positive experience” (a projects staff member); “I was grateful for the time all the consumers put into working on the project” (researcher).

### Potential challenges for consumer involvement

Challenges encountered in the process of working towards increasingly meaningful consumer involvement included: the time commitment required, the lack of diverse communities in the consumer cohort, the entrenched power-dynamics between the doctor/researcher and the patient/consumer, and the reality of challenges associated with living with cancer that resulted in some consumers needing to take a break from their role in the project.

#### Time commitment

Consumers, project staff, and the researcher alike commented on the time commitment required to have meaningful consumer involvement: “more opinions did make it a longer process” (researcher); “we would more or less decide on something but then further thought over time caused changes…there will always be changes that could be made but a time comes when final decisions are made and completion is necessary” (a consumer). Where opinions differed, the decision was made by consensus or informal voting. This ensured that the decisions made reflected the view of the majority of the group, not simply the loudest voice or the most confident contributor. The team also acknowledged that this time was valuable and added significantly to the success of the project and the success of the relationship: “the outcome was ultimately a better care plan” (researcher); “my time was valued” (a consumer). Multiple team members commented on the impact that having regular meetings that ran to time and which followed a clear agenda had on project success so that the most could be made of the time the team spent actively working together. The collaboration between the consumers, project staff, and the researcher, and the development of trusting and meaningful relationships not only contributed value to the outcome of the project but can also be viewed as a valuable outcome in itself. The team created through working together on this project is an exciting foundation for future work: “I am interested in continuing with these programs as a consumer” (a consumer).

#### Power dynamics

While none of the consumers reflected explicitly on the impact of a power dynamic on the functioning of the team and the success of consumer involvement, it was evidently front of mind for the researcher on the project who commented: “I worried that because I’m an oncologist the consumers weren’t always honest with me”. The impact of such a power dynamic may be reflected in the lack of confidence that some consumers reported feeling at the beginning of the project. Being conscious of this imbalance of power, the researcher and project staff were able to make every effort to make discussions inclusive and the research process transparent. Comments from the team reflect this: “the interaction was largely positive, leading to the formation of a strong and engaged team where the hope is that everyone feels able to contribute” (a project staff member). Moving forward, encouraging open conversations about what makes consumers feel more comfortable and confident when working with clinicians will allow our group to continue navigating how to work as an ‘equal team’.

#### Diversity and representation

Members of the team reflected on the difficulty of capturing the diversity of the patient and consumer population in a small consumer team. One consumer noted that “though very difficult, building a more diverse group of consumers may have offered up some different ideas. Younger consumers may have different thoughts on the care plan and its usefulness for example”. This reflection on the lack of representation of diverse communities in our team has acted as a prompt for us to consider ways in which we can enable feedback and input from a broader, more diverse range of consumers.

#### Taking a break

On consumers having to leave the team or suspend their involvement for health reasons or caring responsibilities, the researcher offered “it was very hard when patients relapsed and had to leave the group to have further treatment. It is a tough reminder that we are working with and for cancer patients”. Though tough, this was considered an important reminder that while consumers are central to research, which spans from ‘bedside to bench-side and back again’, their medical needs can compromise their role. Having a team of consumers involved in this research project has helped to alleviate consumer fatigue with regards to workload.

## Discussion

The use of Gibbs’ Reflective Cycle has produced structured reflections from our team and allowed for the identification of strong themes relating to the consumer involvement in the RECAP project. It encouraged critique of the team structure and workings, which may otherwise not be recognised. Previous research by Mathie et al. [2] has suggested the shortcomings of conducting evaluations of consumer involvement without an adequate framework to measure the effect of different approaches. In this research using Gibbs’ Reflective Cycle, we have sought to establish, at least for our team, a useful and re-useable framework that allows for the recording and reporting of consumer involvement in an ongoing research project. This method, along with this article, also seeks to address the issue of the ‘invisibility’ of consumer involvement that Mathie et al. identifies, whereby the practical details of consumer input in a project are rarely available in publicly accessible research documents. Most usefully, as a cyclical process, Gibbs’ Reflective Cycle will also allow us to respond as a team to the ideas and issues identified; for example, recruiting a more diverse consumer group, and, therefore, is a framework that allows for reporting, reflection, and growth.

The reflection conducted by our team using Gibbs’ Reflective Cycle supports published work by Tanay et al. [16] by suggesting that even those who lacked understanding of working with consumers at the beginning of the project were able to gain useful insights through meaningful collaboration. Our findings aligned with those of Tanay et al. as some team members reflected that consumer involvement seemed like a “hurdle” at the beginning of the project, but came to be recognised as an aspect of the research that is “valuable and can enhance a project”. Tanay and colleagues went beyond the use of reflective questionnaires, utilising written records and emails to augment the understanding of a team’s collaboration. Accessing such additional sources for our reflection on consumer involvement in the RECAP project may have added depth to our data.

Within the RECAP project the key document is a personalised care plan, designed for patient education. As it is a patient facing document this is an ideal project to incorporate consumer input and tangibly see the outcomes. Other projects (such as those which may be pre-clinical, for example bench/lab research) may face different challenges in involving consumers meaningfully in their work. The researchers in our team work across many fields of cancer research, from basic science research to Phase III trials. The meaningful consumer involvement in this project and the knowledge gained through collaboration has both improved the capacity of the researchers to engage with consumers and increased their confidence in the process of consumer involvement. For this team, this will encourage consumer involvement in increasingly varied projects, with the aim that, over time, consumers are routinely meaningfully involved in all types of cancer research and in all stages of this research.

As a tool for other research groups seeking to increase consumer involvement in their projects by moving towards a co-design strategy, key learnings from this reflective cycle are summarised in the table below (Table 2).

**Table 2:** Practical learnings

Domain	Learnings
Preparation	A vision statement to set up expectations for the group and establish what consumer involvement will look like
	A clear agenda for each meeting, to be discussed at the end of the preceding meeting
	Documents to be available for an appropriate amount of time pre-meeting, so that everyone has the opportunity to prepare
	Allow for in person and virtual meetings for enhanced accessibility
Meeting structure	Regular meetings that run to time
	Consistent communication around the project or any team updates
	Clearly identified host/facilitator of the meeting
Team culture	Expectation setting at the beginning of the project and revisited throughout
	Commitment to addressing existing power dynamics so that the group can act as an “equal team”, where everyone feels respected and able to contribute
	Having experienced consumers in the group to help guide the consumer interactions
	Institutional support, such as having a Consumer Manager and consumer co-ordinator and remunerating consumers for their involvement, to ensure that consumers are appropriately supported throughout their involvement

## Limitations

Our article is not without limitations. Over the course of the project, we have had eight consumers participating but at the time of completing the reflection we had only four active consumers. While the participants who completed the reflection were part of the project from the early stages, having additional participants’ input would have added depth to our thematic analysis. We have had to acknowledge the difficulty of the loss of team members and that the heartbreaking reality of working with patients with lived experience of cancer is the possibility of further illness and caring responsibilities.

Another limitation of our study is that team members completing the reflective questionnaire were conscious that it would be analysed by the project staff and the researcher. Although the responses were de-identified prior to analysis there was possible bias introduced, with a potential reluctance to feedback negative aspects of the project. We presented only thematic analysis and de-identified quotes to the group to maintain anonymity but, when the reflective cycle is repeated in the future, we plan to have external data identification to reduce this possibility of bias.

## Conclusion

Cancer research is imperative to improving outcomes for patients; partnering with consumers in research projects ensures that the patient and public perspective is at the centre of research priorities, design, interpretation, and dissemination. Working as part of an equal team can be rewarding not only for the outcome of the research project but also in terms of the experience of individual team members. Through reflection on our experience of moving towards co-design, our team has developed and identified practical strategies that contributed to the success of our partnership, and reviewed the areas in which we can improve the way we partner with consumers. These practical reflections on creating a safe and supportive environment in which genuine consumer involvement can take place may help guide other researchers looking to work meaningfully with consumers in research.

## Data Availability

Data sharing is not applicable to this article as no datasets were generated or analysed during the current study.
